# Rehabilitation of the Completely Edentulous Mandible by All-on-Four Treatment Concept: A Retrospective Cohort Study with Up to 10 Years Follow-Up

**DOI:** 10.3390/medicina58010010

**Published:** 2021-12-22

**Authors:** Tommaso Grandi, Luca Signorini

**Affiliations:** 1 Independent Researcher, 20900 Monza, Italy; dott.grandi@libero.it; 2 School of Dentistry, Saint Camillus University of Health Science, 00198 Rome, Italy

**Keywords:** dentistry, stomatology, oral rehabilitation

## Abstract

(1) *Background and Objectives*. Currently, there are no definitive long-term data about clinically significant difference in the failure of prosthesis and implant or marginal bone loss related to the rehabilitation of the completely edentulous mandible by all-on-four treatment concept. The main aim of present investigation was to report the long-term outcomes (10-years follow-up) of complete-arch mandibular rehabilitations based on the all-on-four concept. (2) *Materials and Methods*. Patients in need of extractions of teeth due to the occurrence of caries and/or severe periodontal disease and patients presented with edentulous mandibles were enrolled to the study. A total of 96 participants (mean follow-up period after intervention of 3185.2 days) were enrolled in the study. Participants were evaluated at the first visit, 10 days after intervention and every year after the intervention. Implant and prosthesis survival, bone loss and both local biological and mechanical complications were evaluated during the follow-up period. (3) *Results***.** An implants’ survival rate of 97.9% was observed at the end of the follow-up period. Biological complications were reported in 19.8% of patients, whereas mechanical complications were reported in 27.1% of cases. The average marginal bone level at baseline was −0.03 mm. A significant marginal bone loss was observed after 10-years follow-up (2.5 mm). Binary logistic regression analysis showed significant association between smoke and both marginal bone loss and local biological complications. Lastly, a significant association was observed between bruxism and mechanical complications. (4) *Conclusions*. The high implant and prosthesis survival rate and the moderate incidence of biological and mechanical complications observed in present investigation can be associated to several factors such as high implant primary stability, prosthetic design, and control of the occlusal forces.

## 1. Introduction

The completely edentulous mandible rehabilitation represents one of the most significant oral health care rehabilitation services offered by implant prosthodontics in implant dentistry. Indeed, severe teeth loss affects more than 300 million people in worldwide, also showing an increase in the incidence for each year (3%) [1]. In this context, the implant-supported prosthesis rehabilitation for patients affected by severely atrophic mandible is challenging due to the presence of residual jawbone of very low bone quality [2]. Patients characterized with long-term complete edentulism often show these unique conditions [3,4]. In addition, it is known that posterior mandible progressive bone loss can induce the exposure of the alveolar nerve thus causing pain to patients with complete dentures [5].

In the early 2000s, several authors proposed the use of distally tilted implants as possible solution for these issues thus providing a reliable alternative for patients with severely atrophic mandible [6]. Specifically, distally tilted implants can be used in the mandibular and maxillary posterior portions in absence of bone grafting, with distal implants posterior tilting enabling the use thick bone tissue positioned in the anterior area of maxilla and mandible [7]. This action allows to (a) reduce lengths of cantilever, (b) to broaden the prosthetic base, and (c) to improve implant-to-bone surface regions [8,9]. As concern the number of dental implants to be placed in edentulous jaws, Branemark et al. [10] performed 10-years follow-up survival investigations and found that patients treated with 4 dental implants displayed a higher survival rate respect to those treated with 6 implants. 

The rehabilitation of completely edentulous mandible by using complete-arch fixed prosthesis with a reduced number of tilted implants (*n* = 4) has been further developed applying immediate-function protocols with the connection of the prosthesis in the same day of the surgery [8]. This method is currently called the all-on-four treatment concept [8]. 

Currently, the all-on-four treatment concept is used to exploit the low level of bone tissue generally associated to atrophic jaws thus allowing the immediate function of the prosthesis [11,12,13]. Also, the application of the all-on-four treatment concept allow to avoid tissue regenerative procedures that are related to an increase in costs and morbidity [14,15]. The most common all-on-four treatment concept clinical protocol involves the use of 4 dental implants in the anterior part of complete edentulous jaws, to support a provisional, fixed and immediately loaded prosthesis [14,15,16]. 

The all-on-four treatment concept confirmed to be a useful treatment protocols for patients in need of a complete-arch rehabilitation. Indeed, its application showed great results in the short-, medium- and long-term outcomes [8,17,18]. Patients treated with the all-on-four concept protocol, could suffer a significant reduced implants survival due to the occurrence of both biological and technical complications. Nevertheless, rare solid data are reported about long-term outcomes exceeding 5–7 years in full-arch rehabilitation of patients treated by all-on-four concept. Therefore, long-term follow-up studies based on large patients’ cohort are needed. 

Therefore, the purpose of the present investigation was to report the long-term outcomes of complete-arch mandibular rehabilitations based on the all-on-four concept method.

## 2. Materials and Methods

### 2.1. Study Protocol

In this retrospective study, patients presenting with mandibular edentulism or those with caries and periodontal disease requiring management that result in their becoming edentulous were retrospectively reviewed. The study was conducted according to the guidelines of the Declaration of Helsinki, and approved by the Ethics Committee of Saint Camillus University (protocol code 010-ODONTO of 10 September 2019) [19]. Each patient was counseled and given a consent form regarding their participation in the present investigation about the experimental protocol, the aim of the study and the existence of possible treatment option. At the end of the first visit, patients enrolled in the study signed an informed consent.

96 patients were enrolled from February 2005 to May 2009 in 2 private clinics, both directed by one of the authors (L.S.); the surgical procedures were also performed by the same dental surgeon (L.S.), while the prosthetic procedures were performed by the same team, made by 2 dentistry and 2 dental technicians. The mean follow-up period was 3185.2 days after intervention. All the study participants (*n* = 96) were evaluated at the first visit (100%), 10 days after intervention and every year after the intervention. 

The exclusion criteria for the study were uncontrolled diabetes, unable to commit to a proper follow-up, general contraindications to implant surgery, oncological treatments in the six months before surgery, patients under treatment with intravenous bisphosphonates, heavy smokers (more than 20 cigarettes per day), and implant placement immediately after extraction in periodontally compromised cases. Other exclusion criteria were related to the overall periodontal condition: the lack of sufficient bone volume and the presence of decayed teeth in the regions interested by the prosthetic rehabilitation were considered absolute exclusion criteria. Medical and oral examination was conducted to all patients to determine angle classification.

The guidelines on clinical research involving human subjects according to the declaration of Helsinki were followed. The study has involved a list of consecutive patients requiring a complete-arch rehabilitation in the mandible, with the all-on-four concept. Anamnestic data were properly recorded at the first visit.

### 2.2. Surgical Procedure

In addition to clinical assessment, the study was also based on radiographic investigations, to evaluate bone height; furthermore, computerized tomography scans were performed to assess both the bone volume and the position of specific anatomical structures as the inferior alveolar nerve.

Surgical protocol was performed based on previous studies [20]. Briefly, the preparation of the implant site was achieved considering the density of the bone in order to reach primary stability (30 ≤ x ≥ 80 Newton Centimeter [Ncm] final torque value), the patients were anesthetized with Articaine with adrenaline (1:100,000). Thereafter, the residual teeth were extracted: the surgical technique was extremely conservative, to maintain the alveolar bone walls integrity, and the granulation tissues removed. To detect possible occurrence of bone dehiscence, the alveolar bone was carefully checked for its integrity, by using a periodontal probe. Then, relieving incisions on the buccal aspect in the molar area were performed in order to raise a muco-periosteal flap at the level of the ridge crest. According to the surgical guidelines, sites of the implants and the ideal angulations were identified. All patients enrolled in present investigation were treated with 4 tapered implants (JDEvolution, JDentalCare, Modena, Italy) with internal connection and double acid etched treated surface. As concern the distal implants, 2 tilted implants were placed. Surgeon planned and used the best implant lengths (10 to 15 mm) and diameters (3.2 to 5 mm) (Table 1), based on the patients’ anatomic characteristics, and according to the clinical indications. The cantilever has been reduced by inserting the drill to obtain a more posterior implant position and in correspondence of the mental foramen (crestal side). Also, the implants in distal segments were tilted to achieve the second premolar in the surgical guide. Then, the surgeon inserted the 2 mesial implants. Granules of inorganic bovine bone (0.25 to 1 mm) (Geistlich Bio-Oss, Geistlich Pharma AG) were used to fill gaps larger than 2 mm between implants and the surrounding bone when present. In order to obtain the best implant stability, the osteotomy was undersized. A calibrated torque wrench (JDTorque, JDentalCare) was used to measure the final insertion torque. Conical abutments were connected to the implants. An optimal prosthetic screw access on the distal implants required abutments with an inclination of 30 degrees relative to the implant axis whereas standard or 17-degree abutments were placed on the mesial implants. The abutment screws were tightened at 30 Ncm. After reapproximating the soft tissue healing caps were positioned over the conical abutments. Implants’ characteristics, as well as the torque values, are reported in Table 1.

**Figure 1:**
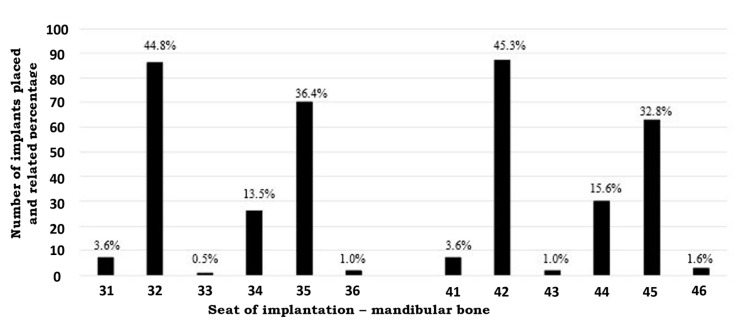
Percentage of Implants placed in teeth sites on mandibular bone.

Figure 1 showed both the percentage and position of implants placed in present investigation.

### 2.3. Prosthodontic Procedure

A 10-unit screw-retained complete-arch fixed denture was manufactured by the same dental laboratory and provided to the patient during the first days after surgery (24–48 h). In order to safely splint the implants and also to reduce the risk of fracture on the provisional restorations, the screw-retained dentures were cast in a non-precious alloy. Afterwards, the teeth made of polymethylmethacrylate (PMMA) were placed and the prostheses screwed by using a torque of 15 Ncm onto the conical abutments. All the prosthetic lateral contacts were checked. A protocol of home oral care and a light diet were recommended to each patient. Six months after the surgery, the definitive metal-resin prostheses were provided. 

### 2.4. Follow-Up Visits and Outcomes

Oral hygiene instructions and diet indications were provided to each patient, including oral hygiene instructions. Follow-up visits have been planned after 10 days, and every single year after surgery for 10 years. 

The implant success was considered the primary outcome for present investigation. 

The following criteria were used to assess the implant success: 

The implant support function has been successfully tested;

The implant has been successfully tested for the stability (both manually and individually); 

Absence of infection sites associated with the implant outcomes; 

Absence of areas characterized by radiolucent aspect next to the implants; 

No esthetic issues highlighted by patients or Prosthodontists;

Implant-supported fixed prosthesis that improved the patient’s comfort and also a satisfactory hygienic maintenance. 

The implants that needed to be removed were classified as “failure”.

The secondary outcomes were:

The marginal bone loss (MBL) evaluated at 5, 7 and 10-years after surgery;

The incidence of both local mechanical and biological complications.

Periapical radiographs were performed at T0 (the day of surgery) and 5, 7 and 10-years of follow-up by using parallel technique with a film holder (Super-Bite, Hawe Neos Dental, KerrHawe Ltd., Lugano, Switzerland). The MBL evaluation were performed on both the distal and mesial areas. Average values were calculated. The level of the marginal bone evaluated at 5, 7 and 10-years after surgery were compared with the values assessed at the day of surgery. This analysis allowed to obtain the MBL value. The following complications were classified as local biological complications: 

fistulae formation; 

peri-implant disease; 

presence of peri-implant pockets ≥ 5 mm; 

bleeding on probing;

clinical attachment loss with concurrent presence of MBL;

local osteoporosis.

The following complications were classified as local mechanical complications: 

loosening of any prosthetic component,

fracture of any prosthetic component.

### 2.5. Statistical Analysis

Multivariate logistic regression analysis was performed to study risk factors related with MBL > 3.0 mm at the last follow up, the occurrence of both mechanical and biological complications, adjusting for confounding: candidate variables were included if significant on univariate analysis or clinically relevant.

## 3. Results

A total of 96 patients (mean age at intervention 66.0 ± 7.2 years, range 42–85 years) were included in the study, with a mean follow-up period after intervention of 3185.2 days (8.7 years, range 2602–4007 days) (Median: 3125 days, 8.6 years). All the participants (*n* = 96) were evaluated at the first visit (100%) after intervention and every year after the intervention. There were very few patients lost to follow up (*n* = 10 (10.4%), *n* = 8 lost after a mean of 1.8 years for consent withdrawal, *n* = 2 died for a myocardial infarction 4 and 12 months after placement). The last censored follow-up was 31 December 2020. About the 70% of patients were non-smokers whereas only the 6.3% smoked more than 10 cigarettes/day. The most common co-morbidities were hypertension (34.9%) and diabetes (10.4%). On note, about 6% of patients were osteoporotic at the time of the first visit. As opposing dentition, about half of the patients showed fixed arch on implants (59.4%); the other patients had natural teeth. Baseline patients’ characteristics are reported in Table 2.

Outcomes: As concern the implants’ survival, a success of 97.9% were observed at the end of follow-up period. Indeed, the failure of only 2 implants were noted. In these patients, the implants were repositioned, and the prosthesis replaced.

During the follow-up, biological complications were reported in 19.8% of patients. Specifically, bleeding on probing were observed in 14.6% of patients at 3.6 ± 0.8 years after the procedure, whereas mucositis was detected in 5.2% of subjects at 8 and 16 months, 2, 4 and 5 years respectively. Mechanical complications were reported in 27.1% of cases. 

**Figure 2:**
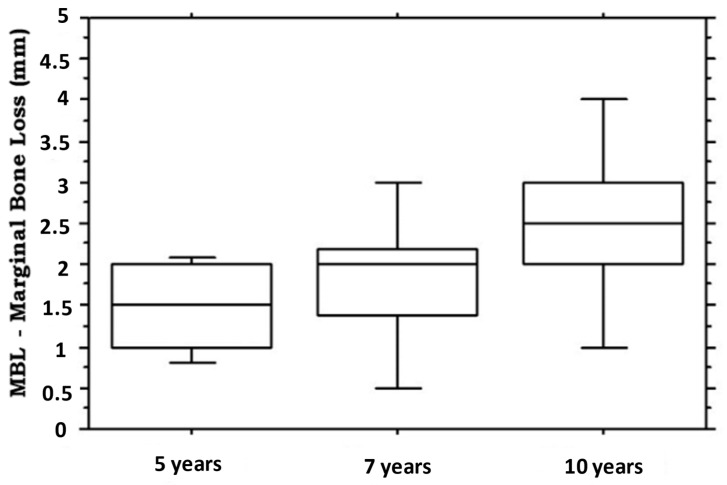
Boxplot illustrating the marginal bone loss measured in millimeters at 5-, 7- and 10-years of follow-up. The median is the horizontal black line inside the box.

The average marginal bone level at baseline was −0.03 mm. The average (95% confidence interval) MBL was 1.5 mm (95% CI: 1.3, 1.7; range: 0.5–2.2) at the 5-year (Figure 2). At the 7-year follow-up, the average MBL was 1.8 mm (95% CI:1.3, 2.3; range: 0.1–3.2) (Figure 2). At the 10-year follow-up, the average MBL was 2.5 mm (95% CI:1.7, 3.3; range: 0.9–5.1) (Figure 2).

Binary logistic regression analyses were performed to evaluate the potential risk indicators for marginal bone loss > 3.0 mm (Table 3), the incidence of biological complications (Table 4) and the incidence of mechanical complications (Table 5) at the last follow up. Binary logistic regression analysis showed a significant association between smoke and both a marginal bone loss > 3.0 mm and the incidence of biological complications. The occurrence of biological complications was also significantly associated to a marginal bone loss > 3.0 mm. As expected, the bruxism was significantly associated to the incidence of mechanical complications. 

## 4. Discussion

Patients with edentulous mandible frequently incur in the post-operative complications after the placement of dental implants. Thus, the development of new rehabilitation techniques such as the all-on-four treatment concept are needed to improve the management of these patients. 

In this context, implant-supported prostheses and/or implant-retained frequently provide patient satisfaction especially if compared to the historical removable denture treatments [21,22,23]. All-on-four technique allow to avoid bone grafting procedures, as well as to use immediately loaded provisional prosthesis by applying four implants in the anterior part of an edentulous arch [24,25]. As already confirmed and reported in the current literature, this protocol can provide a reliable opportunity to oral rehabilitation of the edentulous jaw [26,27,28]. Nevertheless, long-term follow-up data are even more necessary to establish the effective clinical success of the all-on-four treatment concept in patients with edentulous mandible.

Thus, the main aim of present investigation was to report the long-term outcomes of complete-arch mandibular rehabilitations based on the all-on-four concept protocol. To this end, 96 consecutive patients underwent to complete-arch mandibular rehabilitations through the all-on-four concept technique were included in present investigation. The mean follow-up period after intervention was 3185.2 days (with up to 10 years follow-up).

Data reported in this study indicate that this technique can lead to an excellent prognosis at least for 10 years of function. In fact, present investigation assessed the 10-years outcomes of the all-on-four concept for full-arch rehabilitation of the mandible demonstrating a 97.9% cumulative implant success rate.

Biological complications, such as bleeding on probing [29] were reported in 19.8% of patients, whilst mechanical complications were observed in 27.1% of cases. These data are better than what has been reported in the main literature on this matter [29,30,31,32].

Binary logistic regression analyses were performed to study the risk factors related to both mechanical and biological complications. Interestingly, smoke was associated to both the MBL and the occurrence of biological complications. As concern the marginal bone loss, the association between smoke and bone metabolisms has been known for a long time [30]. In particular, smoke is currently recognized as a risk factor for osteoporosis and associated to a significant reduction of the activities of the osteoblasts [31,32]. Similarly, smoking could be associated with some of the observed biological complications as bleeding [31,33]. In line with these data, a significant association was also found between the incidence of biological completions and the loss of marginal bone. 

In addition, it is not surprising the association between bruxism and the incidence of mechanical complications. In fact, the continuous stress forces applied on the prosthesis in patients affected by bruxism can be related to the loosening or the fracture of any prosthetic component [34]. The opposing dentition to dental implant is also important to be carefully considered: in fact, removable prostheses were reported to be severe risk factors to late failure [34]. Basically, the difficulty in a proper distribution of masticatory loads on the removable denture could represent a risk factor that would require meticulous occlusal adjustment for an implant long-term survival. 

The high implant and prosthesis survival rate and the moderate incidence of biological and mechanical complications observed in present investigation can be associated to several factors such as high implant primary stability, prosthetic design, and control of the occlusal forces. Moreover, the immediate load force may contribute to the bone regeneration, thus improving the stability of both implants and prosthesis. 

## 5. Conclusions

The development of full-arch rehabilitation techniques is one of the hot topics of the dental research [35,36,37,38]. Data of present investigation allow to conclude that the full-arch rehabilitation of the edentulous mandible ad modum all-on-four is a reliable treatment option for the rehabilitation of the completely edentulous mandible. However, it is important to remember that biological and mechanical complications can occur. Several conditions may affect the success of the implant-supported rehabilitations such as an MBL > 3 mm, the implant failure, bruxism, smoking and the biological complications. Thus, the stratifications of patients based on anamnestic and/or clinical data can improve the management of patients with edentulous mandible by both increasing the implant success rate and decreasing biological and mechanical complications. 

## Figures and Tables

**Table 1:** Implants characteristics and torque values.

Diameters	Length	Insertion Torque (Ncm)	
3.2 mm: 18 (4.7%)	10 mm: 11 (2.9%)	30 *n*: 7 (1.82%)	50 *n*: 3 (0.78%)
3.7 mm: 300 (78.1%)	11.5 mm: 82 (21.4%)	35 *n*: 6 (1.56%)	60 *n*: 70 (18.22%)
4.3 mm: 65 (16.9%)	13 mm: 221 (57.6%)	40 *n*: 1 (0.26%)	70 *n*: 2 (0.52%)
5.0 mm: 1 (0.3%)	15 mm: 70 (18.1%)	45 *n*: 23 (5.99%)	80 *n*: 272 (70.8%)

**Table 2:** Patients’ baseline characteristics.

Patients *n* = 96	
**Age (years)**	66.0 ± 7.2 (range 42–86 years)
**Male/Female**	39 (40.6%)/57 (59.4%)
**Smoke**	No: 70 (72.9%) <10 cigarettes/day: 20 (20.8%) >10/<20 cigarettes/day: 6 (6.3%)
**Initial situation**	Edentulous: 15 (15.6%) Terminal dentition: 81 (84.4%)
**Co-morbidities**	Hypertension 33/96 (34.9%) Controlled Diabetes 10/96 (10.4%) Hyperthyroidsm 7/96 (7.3%) Osteoporosis 6 (6.3%)

**Table 3:** Risk indicators for marginal bone loss > 3.0 mm (OR stands for Odds Ratio, while CI stands for Confidence Interval, *p* < 0.05 was considered statistically significant, we replicate the test for significant results “a”).

Factor	OR (95% CI)	*p*	OR ^a^ (95% CI)	*p*
Age	1.0 (0.9–1.1)	0.92		
Smokers	7.3 (2.0–27.1)	0.003	9.9 (2.3–43.9)	0.002
Mechanical Complications	1.4 (0.4–5.1)	0.60		
Biological Complications	10.4 (5.1–20.2)	0.001	13.4 (6.3–17.2)	0.0001
Opposing Dentition	1.7 (0.8–5.2)	0.34		
Systemic conditions	0.5 (0.2–1.8)	0.31		

**Table 4:** Risk indicators for Biological Complications (OR stands for Odds Ratio, while CI stands for Confidence Interval, *p* < 0.05 was considered statistically significant, we replicate the test for significant results “a”).

Factor	OR (95% CI)	*p*	OR ^a^ (95% CI)	*p*
Age	1.0 (0.9–1.1)	0.47		
Smokers	7.7 (2.6–23.1)	0.0003	11.3 (3.2–39.7)	0.001
Mechanical Complications	1.3 (0.4–3.9)	0.62		
Opposing Dentition	1.5 (0.6–4.0)	0.76		
Systemic conditions	0.8 (0.3–2.6)	0.80		

**Table 5:** Risk indicators for Mechanical Complications (OR stands for Odds Ratio, while CI stands for Confidence Interval, *p* < 0.05 was considered statistically significant, we replicate the test for significant results “a”).

Factor	OR (95% CI)	*p*	OR ^a^ (95% CI)	*p*
Age	1.0 (0.9–1.1)	0.89		
Smokers	1.6 (0.6–4.4)	0.31		
Biological Complications	1.3 (0.4–3.9)	0.62		
Bruxism	8.7 (2.4–13.8)	0.001	7.9 (1.7–36.6)	0.008
Opposing dentition	1.3 (0.4–3.9)	0.66		
Systemic conditions	1.5 (0.5–4.3)	0.42		

## Data Availability

Raw data are available at the office of prof. Signorini (L.S.).
